# The Increased Expression of Connexin and VEGF in Mouse Ovarian Tissue Vitrification by Follicle Stimulating Hormone

**DOI:** 10.1155/2015/397264

**Published:** 2015-10-11

**Authors:** Yanzhou Yang, Jie Chen, Hao Wu, Xiuying Pei, Qing Chang, Wenzhi Ma, Huiming Ma, Changchun Hei, Xiaomin Zheng, Yufang Cai, Chengjun Zhao, Jia Yu, Yanrong Wang

**Affiliations:** ^1^Key Laboratory of Fertility Preservation and Maintenance of Ministry of Education, Key Laboratory of Reproduction and Genetics in Ningxia, Department of Histology and Embryology, Ningxia Medical University, Yinchuan 750004, China; ^2^Department of Human Anatomy, Inner Mongolia Medical University, Hohhot 010010, China

## Abstract

Ovarian follicular damages were caused by cryoinjury during the process of ovarian vitrification and ischemia/reperfusion during the process of ovarian transplantation. And appropriate FSH plays an important role in antiapoptosis during ovarian follicle development. Therefore, in this study, 0.3 IU/mL FSH was administered into medium during mouse ovarian cryopreservation by vitrification to ascertain the function of FSH on ovarian vitrification and avascular transplantation. The results suggested that the expressions of Cx37, Cx43, apoptotic molecular caspase-3, and angiogenesis molecular VEGF were confirmed using immunohistochemistry, western blotting, and real-time PCR, and the results suggested that the treatment with FSH remarkably increased the number of morphologically normal follicles in vitrified/warmed ovaries by upregulating the expression of Cx37, Cx43, VEGF, and VEGF receptor 2, but downregulating the expression of caspase-3. In addition, the vitrified/warmed ovaries were transplanted, and the related fertility was analyzed, and the results suggested that the fertility, neoangiogenesis, and follicle reserve were remarkably increased in the FSH administrated group. Taken together, administration of 0.3 IU/mL FSH during ovarian cryopreservation by vitrification can maintain ovarian survival during ovarian vitrification and increases the blood supply with avascular transplantation via upregulation of Cx43, Cx37, and VEGF/VEGFR2, as well as through its antiapoptotic effects.

## 1. Introduction

Recent studies have suggested that the prevalence of cancer in females has increased by 20%, and a trend towards younger women has also been observed [1]. Radiotherapy and chemotherapy are the main methods for cancer treatment, and 90% of children and adolescent patients with cancer have hope for a cure [2, 3]. However, the risk of ovarian damage and infertility is present, particularly reduction of the primordial follicle reserve, which may trigger POF (premature ovarian failure) [4, 5]. Thus, cryopreserved-thawed ovarian tissue and transplantation act as an important method to preserve ovarian function during radiotherapy and chemotherapy, and ovarian cryopreservation by vitrification is a very effective and extensively used method to cryopreserve ovaries [6–8].

However, due to cryoinjury in ovarian tissue during vitrification and the possibility of follicular developmental delay and partial apoptosis [9–11] as well as the fact that most ovarian follicles die from ischemia/reperfusion injury in the early stage of transplantation [12], neoangiogenesis is indispensable and increases around the transplanted ovary within 48 h [13, 14] to protect the survival of the ovarian follicle. Hence, the decreasing of follicle die and increasing the neoangiogenesis are two important objects in ovarian vitrification and transplantation.

Previous studies revealed that FSH (follicle-stimulating hormone) plays an important role in the growth and development of follicles, particularly in the antiapoptosis of the ovarian granulosa cell [15–17]; therefore, granulosa cell apoptosis is inevitable in the absence of FSH during ovarian vitrification* in vitro*. Previous studies have revealed that granulosa cells are essential for growth and development of the follicle [18] and that follicular atresia is triggered by granulosa cell apoptosis; connexin expression is negatively correlated with follicle apoptosis [19] during granulosa cell development, which occurs around immature follicle development. FSH can enhance the expression of connexin [20] via indirect inhibition of the activation of the antiapoptotic protein. In addition, ovarian angiogenesis and the expression of VEGF are regulated by FSH [21–23].

Our previous studies have suggested that administration with 0.6 IU/mL HMG (0.3 IU/mL FSH and 0.3 IU/mL LH) in ovarian culture* in vitro* remarkably improves the blood supply reconstruction with avascular transplantation and does not cause excessive ovarian follicle activation and depletion [24].

Thus, we proposed that FSH might play an important role in preserving ovarian survival during cryopreservation by vitrification and avascular transplantation; hence, in this study, 0.3 IU/mL FSH was administrated into vitrification solution and the function of FSH was explored in the ovarian vitrification and transplantation.

## 2. Materials and Methods

### 2.1. Ethics Statement

The Committee for the Ethics on Animal Care and Experiments in Ningxia Medical University approved the study protocol. All operations on animals were performed under sodium pentobarbital anesthesia, and all efforts were made to minimize suffering.

### 2.2. Animals and Treatments

Four-week-old C57BL/6J mice were purchased from Jackson Laboratories (United States, ID number: 000664) and were maintained at 24 ± 2°C in a light-controlled room (12 h light : 12 h darkness) with free access to food and water. The estrous cycle was tracked by vaginal smear according to previous studies [25–27], and mice with diestrus were used for this study.

### 2.3. Experimental Grouping and Protocol

The sacrificed mice and the collection of ovaries are described below. Briefly, mice with diestrus were anesthetized with sodium pentobarbital, hair on the back was removed with a razor, the muscle layer was incised with surgical scissors, and the ovaries were exposed and collected. All procedures were performed under aseptic conditions. A total of 100 ovaries were collected from 50 mice and divided into five groups with 20 whole ovaries in each group. The groups were divided as follows: (A) control group (CG): fresh ovaries were collected from the mice and immediately fixed in 4% paraformaldehyde for immunohistochemistry and other ovaries were preserved in liquid nitrogen for RNA and protein extraction; (B) NG-FSH: the ovaries underwent vitrified/warmed process without any further treatment; (C) OG-FSH: 0.3 IU/mL FSH was administered into the medium during the entire vitrification/warming process; (D) EG-FSH: 0.3 IU/mL FSH was administered into the medium during the early process of vitrified cryopreservation, which contains preculture, preequilibrium, and osmotic equilibrium; (E) LG-FSH: 0.3 IU/mL FSH was administered into the medium during the late process of vitrified cryopreservation, which consists of warming and postculture with culture solution.

#### 2.3.1. Vitrified Cryopreserved Procedure

The preparation of the basic medium, culture, freezing, and thawing solution are described in supplemental doc1 in Supplementary Material available online at http://dx.doi.org/10.1155/2015/397264, and the vitrification procedure was performed. Briefly, the process mainly consists of preincubation in culture solution for 1 h at 37°C with 5% CO_2_. The whole ovary was preequilibrated for 8 min with preequilibration solution, and then osmotic equilibrium was achieved for 3.5 min with incubation in the vitrified solution. The whole ovary was preserved in liquid nitrogen for at least one day, warmed for 10 min with a gradient thawing solution, and postcultured for 1 h at 37°C with 5% CO_2_ with culture solution.

### 2.4. RNA Extraction and cDNA Synthesis

Total RNA was extracted using TRIzol reagent (TaKaRa, Dalian, China) according to the manufacturer's instructions. Extracted RNA was dissolved in diethylpyrocarbonate- (DEPC-) treated water, and the RNA concentration and purity were estimated at 260 and 280 nm using a spectrophotometer (Eppendorf, Inc., Hamburg, Germany). The absorption (260/280 nm) ratios of all preparations were between 1.8 and 2.0. Aliquots of RNA samples were subjected to electrophoresis using a 1.2% agarose gel with ethidium-bromide staining to confirm RNA integrity. The cDNA was reverse-transcribed using the PrimeScipt RT reagent kit (TaKaRa, Dalian, China) according to the manufacturer's instructions.

### 2.5. Real-Time Quantitative Polymerase Chain Reaction (RT-qPCR)

Real-time quantitative PCR was performed using 7500 Software v2.0.5 (7500 Fast Real-Time PCR System, ABI, USA). The GenBank accession number of the mRNA, the primer sequences, and the annealing temperatures are listed in Table 1. The mouse GAPDH gene, which was used as a reference gene, was amplified in parallel with the target gene, allowing gene expression normalization, and 2^−ΔΔCt^ was used for quantification. The RT-qPCR product was obtained using SYBR Premix Ex Taq II (TaKaRa, Dalian China) according to the manufacturer's instructions. Each PCR reaction was performed in a 20.0 *μ*L reaction mixture containing 10.0 *μ*L of 2× SYBR Premix Ex Taq II, 2.0 *μ*L of cDNA (the equivalent of 20 ng of total RNA) as the template, 0.8 *μ*L aliquots of each primer at 10 *μ*M, and 6.4 *μ*L of nuclease-free water. The PCR cycling conditions consisted of one cycle at 95°C for 30 s, followed by 40 cycles at 95°C for 5 s and finally 60°C for 30 s. The experiments were performed in triplicate for each data point, and the mean of these values was used for the final analysis.

### 2.6. Immunohistochemistry

Immunohistochemistry was performed according to previous methods [28, 29]. Briefly, after dehydration, antigen retrieval was performed in citrate buffer (pH 6.0) by treating the samples twice in a microwave oven at 100°C for 15 min; then, the slides were washed three times with PBS. The sections were pretreated with 0.3% (v/v) H_2_O_2_ in methanol to quench endogenous peroxidase activity. After being washed with PBS, the sections were incubated with 10% goat serum for 30 min at 37°C. Following blocking, sections were incubated overnight with rabbit anti-human polyclonal Cx43 antibody (ab11370, 1 : 100 dilution, UK), rabbit anti-mouse polyclonal Cx37 antibody (ab58918, 1 : 100 dilution, UK), rabbit anti-human polyclonal active caspase-3 antibody (ab2302, 1 : 100 dilution, UK), rabbit anti-human polyclonal VEGF (ab46154, 1 : 100 dilution, UK), rabbit anti-mouse polyclonal CD31 (ab28364, 1 : 100 dilution, UK), and rabbit anti-mouse polyclonal CD34 (ab81289, 1 : 100 dilution, UK) at 4°C; they were then washed with PBS and incubated with biotinylated anti-rabbit or mouse IgG antibody (Beijing 4A Biotech Co., Ltd., Beijing, China) for 1 h at 37°C. Sections were washed 3 times with PBS and then incubated with HRP-labeled streptavidin (SA-HRP) for 30 min at 37°C. Thereafter, positive reactions were visualized with a diaminobenzidine- (DAB-) peroxidase substrate for 30 s and the nuclei were counterstained with hematoxylin. Appropriate negative slides were run in parallel without the addition of primary antibody. Slides were imaged using a digital microscope (BA400, Motic, Wetzlar, Germany).

### 2.7. Western Blot Analyses

SDS (Sodium Dodecyl Sulfate) gel electrophoresis and western blot analyses were performed on homogenates of the ovary samples. To standardize the loading samples, the protein contents of the cleared supernatant of the homogenates were determined using a BCA assay. The samples were then separated on a 12% SDS gel with a 5% stacking gel under reducing conditions and transferred onto polyvinylidene difluoride (PVDF) membranes. The membranes were blocked for 1 h with 5% nonfat dry milk and probed with the primary antibody at a concentration of 1 *μ*g/mL^−1^ for 1 h and then with a streptavidin-horseradish peroxidase-conjugated anti-rabbit antibody (ZhongBin, GOLDEN BRIDGE, China) at a dilution of 1 : 7000. The resulting signal was visualized using the ECL Detection Kit (Thermo) according to the manufacturer's instructions. *β*-actin was used as the reference. These results were analyzed using Image J Software.

### 2.8. Terminal Deoxynucleotidyl Transferase-Mediated dUTP-Biotin Nick End Labelling Assay (TUNEL)

Apoptosis was determined using the in situ terminal deoxynucleotidyl transferase-mediated nick end labelling (TUNEL) assay (Roche Diagnostics, Meylan, France). Next, the sections were washed and double-stained with 1 *μ*g/mL DAPI (4′,6-diamidino-2-phenylindole) at room temperature for 20 min. The positive ovarian cells were examined using fluorescence microscopy, and the apoptotic rate was analyzed using IPP 6.0 Software.

### 2.9. Ovary Orthotopic Transplantation and Quantification of Litter Size

The ovaries from the CG, NG-FSH, and OG-FSH groups were orthotopically transplanted into 8-week-old allogenic female recipient mice of the C57BL/6J-Tyr^c-2J^-J albino strain. There were 10 mice in each group. The recipient female mice underwent oophorectomies prior to transplantation. The ovary orthotopic transplantation procedures were performed as previously described [30]. The recipient mice were mated with proven males of the same strain to induce natural pregnancy at 4-5 days after the emergence of the normal estrous cycle. A vaginal smear was checked on the fifth day, eighth day, eleventh day, fourteenth day, and seventeenth day after transplantation; the average litter size and the average litter size interval, which are two important reproductive traits, were quantified after delivery.

### 2.10. Vascular Perfusion

Half of a whole ovary (volume of 1 mm × 1 mm × 2 mm) was transplanted under the kidney capsule as previously described [31], 2MD-FITC-Dextran (Sigma, FD-2000S) was injected into the tail veins after 48 h of transplantation, and grafts were cut and prepared for frozen sections of 60 *μ*m. The tissue sections were imaged using laser scanning confocal microscopy. Other ovarian grafts were collected, the ovarian follicle was counted, and the neoangiogenesis markers CD31 and CD34 were checked by immunohistochemistry.

### 2.11. Follicle Count

Paraffin-embedded ovaries were cut into serial sections and stained using hematoxylin and eosin (H&E). The ovarian follicles were counted according to previous studies [24, 32].

### 2.12. Statistical Analyses

All experiments were replicated at least three times for each group. The data were presented as the mean ± SEM. Data were analyzed with ANOVA followed by Fisher's Least Significant Difference Test (Fisher's LSD) with SPSS software (Version 13.0; SPSS, Inc., Chicago, IL). Differences were considered significant at *P* < 0.05.

## 3. Results

### 3.1. Morphological Observation of the Ovaries

**Figure 1:**
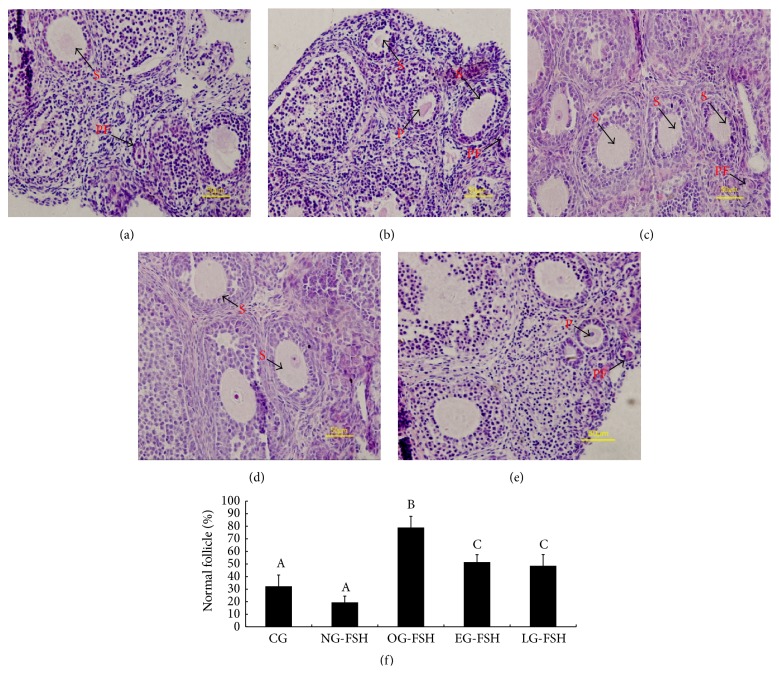
Quantification of the normal follicle. Compared with the CG and NG-FSH groups, the morphological structure of the ovarian tissues in the OG-FSH group maintained its integrity, and the percentage of normal follicles was significantly higher compared to the other groups (*P* < 0.05) (Figure 1). (a)–(e) Results of HE staining. (a) CG (fresh control group). (b) NG-FSH (no FSH was administered during ovarian vitrification). (c) OG-FSH (FSH was administered during ovarian vitrification). (d) EG-FSH (FSH was administered during the early process of ovarian vitrification). (e) LG-FSH (FSH was administered during the late process of ovarian vitrification). PF indicates primordial follicles; P indicates primary follicles; S indicates secondary follicles (S). (f) Quantification of normal ovarian follicles for the different groups. The different letters indicate a significant difference; the same letters indicate no difference.

Compared with the CG and NG-FSH groups, the morphological structure of the ovarian tissues in the OG-FSH group showed integrity, and the percentage of morphologically normal follicles in the OG-FSH group was significantly higher than that in the other groups (*P* < 0.05) (Figure 1). However, the total number of follicles in each group was not statistically significant.

### 3.2. Detection of Apoptosis during Ovarian Vitrified Cryopreservation

**Figure 2:**
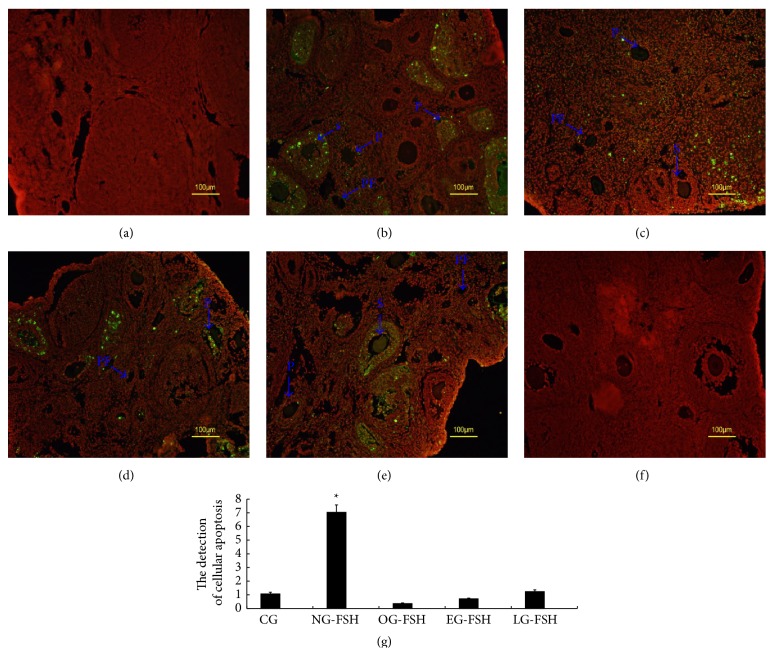
Apoptosis in the different groups using TUNEL. TUNEL results showed that apoptosis mainly occurred in the granulosa cells and oocytes of the primordial follicle, primary follicle, secondary follicle, and antral follicle, with little apoptosis in the primordial follicle. TUNEL results suggested that the ovarian apoptotic rates of CG, OG-FSH, EG-FSH, and LG-FSH groups were significantly lower than that of the NG-FSH group. ∗ indicates a statistically significant difference compared with the other groups. (a) CG. (b) NG-FSH. (c) OG-FSH. (d) EG-FSH. (e) LG-FSH. (f) Negative control. (g) Analysis of apoptotic cell. PF indicates primordial follicles; P indicates primary follicles; S indicates secondary follicles (S).

Apoptosis of the ovarian cell was confirmed using TUNEL, and the apoptosis mainly occurred in the granulosa cell and oocyte of the primary follicle, secondary follicle, and antral follicles. Apoptosis was rarely detected in the primordial follicle. These results suggested that the apoptotic rate in the ovarian cell of CG (Figure 2(a)), OG-FSH (Figure 2(c)), EG-FSH (Figure 2(d)), and LG-FSH (Figure 2(e)) groups was significantly lower than that in NG-FSH group (Figure 2(b)).

**Figure 3:**
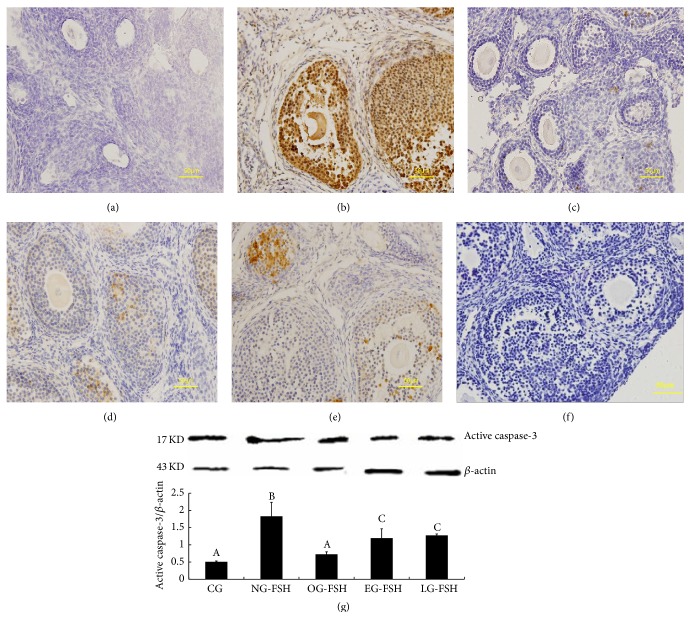
The check of apoptotic marker caspase-3. (a)–(f) Localization of the apoptotic marker active caspase-3 using immunohistochemistry. The apoptotic molecular marker, active caspase-3, was mainly localized in the granulosa cell and oocyte and specifically in the primary follicle, secondary follicle, and antral follicle. (a) CG. (b) NG-FSH. (c) OG-FSH. (d) EG-FSH. (e) LG-FSH. (f) Negative control. (g) Caspase-3 protein expression. Caspase-3 protein expression in the CG, OG-FSH, EG-FSH, LG-FSH, and NG-FSH groups. The different letters indicate significant differences; the same letters indicate no differences.

Consistent with the results from TUNEL, the apoptotic marker, active caspase-3, was also mainly localized in the granulosa cells and oocytes (Figures 3(a)–3(e)), and the levels of protein (Figure 3(g)) in the CG, OG-FSH, EG-FSH, and LG-FSH groups were significantly lower than those in the NG-FSH group.

### 3.3. Expression of Cx43, Cx37, and VEGF/VEGF Receptor 2 (VEGFR2)

**Figure 4:**
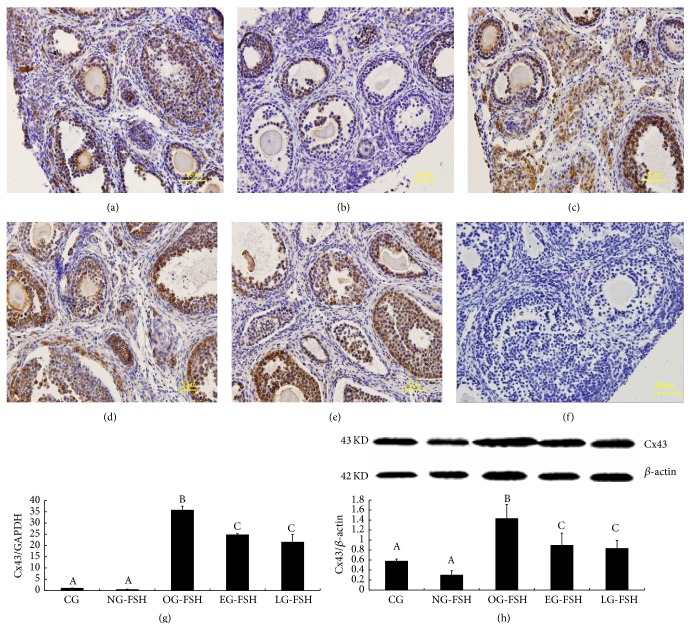
Detection of Cx43 expression. (a)–(f) Localization of Cx43 in the ovarian cell among the different groups. (a) CG. (b) NG-FSH. (c) OG-FSH. (d) EG-FSH. (e) LG-FSH. (f) Negative control. (g)-(h) Cx43 mRNA and protein expression. (g) Cx43 mRNA expression in the CG, OG-FSH, EG-FSH, LG-FSH, and NG-FSH groups. (h) Cx43 protein expression in the CG, OG-FSH, EG-FSH, LG-FSH, and NG-FSH groups. The different letters indicate significant differences; the same letters indicate no differences.

**Figure 5:**
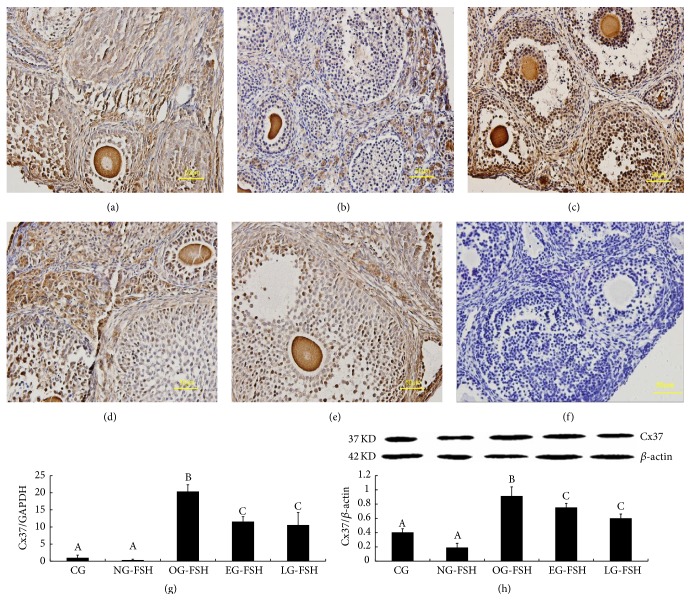
Detection of Cx37 expression. (a)–(f) Localization of Cx37 in the ovarian cell among the different groups. (a) CG. (b) NG-FSH. (c) OG-FSH. (d) EG-FSH. (e) LG-FSH. (f) Negative control. (g) Cx37 mRNA expression in the CG, OG-FSH, EG-FSH, LG-FSH, and NG-FSH groups. (h) Cx37 protein expression in the CG, OG-FSH, EG-FSH, LG-FSH, and NG-FSH groups. The different letters indicate significant differences; the same letters indicate no differences.

Localization of Cx43 and Cx37 was confirmed using immunohistochemistry according to previous studies [28, 29]. Cx43 was mainly localized in the granulosa cell (Figures 4(a)–4(e)), and Cx37 was mainly localized in the oocyte and granulosa cell (Figures 5(a)–5(e)). Furthermore, the expression of Cx43 (Figures 4(g)-4(h)) and Cx37 (Figures 5(g)-5(h)) mRNA and protein in the OG-FSH, EG-FSH, and LG-FSH groups was significantly higher than that in the CG and NG-FSH groups (*P* < 0.05).

**Figure 6:**
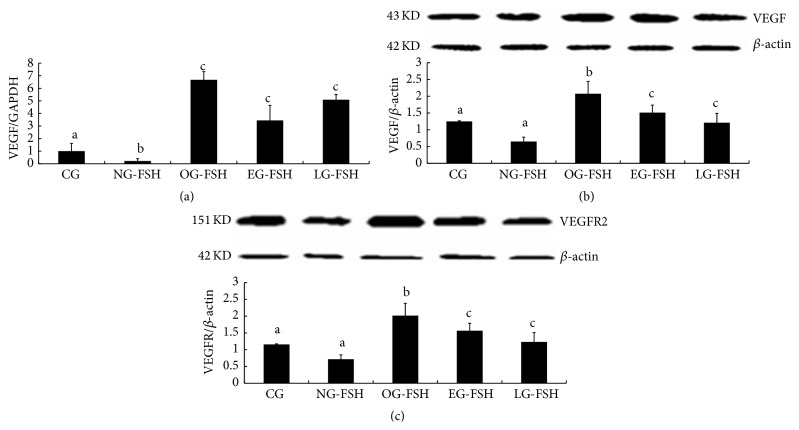
Detection of VEGF mRNA and VEGF and VEGFR2 protein expression. (a) Results of VEGF mRNA expression. (b) Results of VEGF protein expression. (c) Results of VEGFR2 protein expression. The different letters indicate significant differences; the same letters indicate no differences.

In addition, the expression of VEGF mRNA was confirmed and the results suggested that the expression of VEGF in the OG-FSH, EG-FSH, and LG-FSH groups was significantly higher than that in the other groups (*P* < 0.05); and the protein expression of VEGF and VEGFR2 was confirmed, and the results suggested that the expression of VEGF (Figures 6(a)-6(b)) and VEGFR2 (Figure 6(c)) in the OG-FSH group was significantly higher than that in the other groups (*P* < 0.05).

### 3.4. Morphological Observation of the Ovaries 48 h after Transplantation

**Figure 7:**
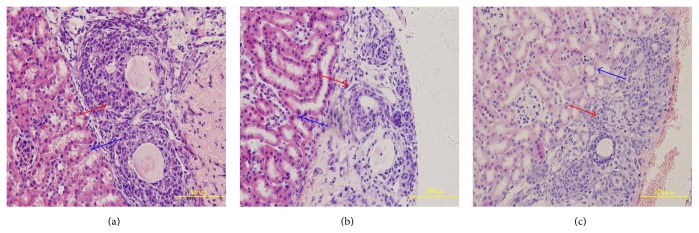
Ovarian morphology 48 h after transplantation. The ovarian morphology of CG and OG-FSH maintained its integrity but the ovarian morphology of NG-FSH displayed scant follicles and scattered ovarian stroma. (a) CG. (b) NG-FSH. (c) OG-FSH. Red arrow indicates the ovarian grafts; blue arrow indicates the renal tissue.

The ovaries of CG, NG-FSH, and OG-FSH were heterotopically transplanted by the kidney capsule (supplemental doc.2a); after 48 h, the ovarian grafts were embedded with paraffin and cut into serial sections and stained using hematoxylin and eosin (H&E), and the results suggested that the ovarian morphology of CG (Figure 7(a)) and OG-FSH (Figure 7(c)) groups maintained its integrity; however, the ovarian morphology of NG-FSH (Figure 7(b)) revealed scant follicles and scattered ovarian stroma.

The ovarian follicle was counted according to previous studies [24, 32], and the percentage of primordial follicles, primary follicles, and morphologically normal follicles (all stage follicles) in the CG and OG-FSH groups was significantly higher than that in the NG-FSH group (*P* < 0.05). The percentage of atretic follicles in the CG and OG-FSH was significantly lower than that in the NG-FSH group (*P* < 0.05) (Table 2).

### 3.5. Confirmation of Neoangiogenesis

**Figure 8:**
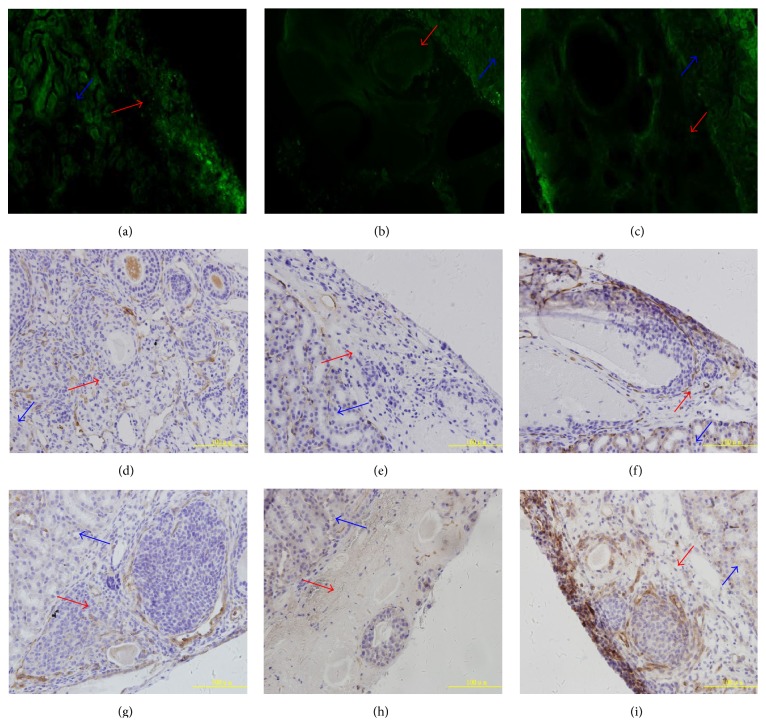
Detection of neoangiogenesis using vascular perfusion. The green fluorescence indicates neoangiogenesis (a) CG. (b) NG-FSH. (c) OG-FSH. (d)–(f) The expression of CD31 in the CG and OG-FSH groups was remarkably increased compared to the NG-FSH group. (d) CG. (e) NG-FSH. (f) OG-FSH. (g)–(i) The expression of CD34 in the CG and OG-FSH groups was remarkably increased compared to the NG-FSH group. (g) CG. (h) NG-FSH. (i) OG-FSH. Red arrow indicates the ovarian grafts; blue arrow indicates the renal tissue.

Consistent with the expression of VEGF and VEGFR2, neoangiogenesis in the CG and OG-FSH groups was remarkably increased compared with the NG-FSH group (Figures 8(a)–8(c)). In addition, the endothelial cell markers CD31 and CD34 were checked by immunohistochemistry, and the results suggested that the expression of CD31 (Figures 8(d)–8(f)) and CD34 (Figures 8(g)–8(i)) in the CG and OG-FSH groups was remarkably increased compared with the NG-FSH group.

### 3.6. Quantification of Litter Size of the Ovarian Transplanted Mice

Ovaries obtained from the CG, NG-FSH, and OG-FSH groups were orthotopically transplanted (supplemental doc.2b). The pregnant female mice were allowed to litter, and the size of each litter was recorded; the pups have black fur (supplemental doc.3: a: CG; b: OG-FSH; c: NG-FSH). After 6 and 12 months, the number of pregnant mice, the pregnancy rate, the average litter size, and the average litter size interval of each group were determined (Tables 3 and 4). In addition, the number of pregnant mice in the CG, NG-FSH, and OG-FSH groups was 5, 3, and 4, respectively, and the pregnancy rate of the CG, NG-FSH, and OG-FSH groups was 50%, 30%, and 40%, respectively. Moreover, the number of pregnant mice and pregnancy rate of the CG and OG-FSH groups were remarkably increased compared with the NG-FSH group. The average litter size in the CG and OG-FSH groups was significantly higher than that in the NG-FSH group (*P* < 0.05), and the average litter size interval in the CG and OG-FSH groups was lower than that in the NG-FSH group (*P* < 0.05).

**Figure 9:**
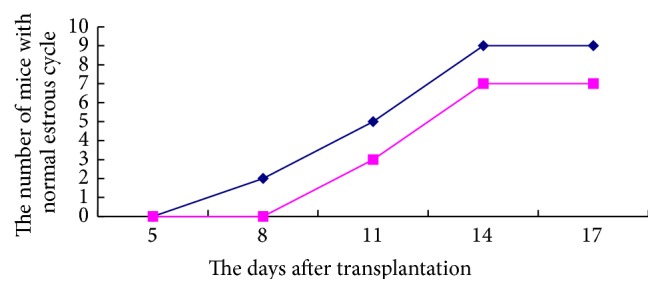
Recovery of the estrous cycle after transplantation. Recovery of the estrous cycle after transplantation in the OG-FSH group was remarkably higher than mice in the NG-FSH group. -⋄- indicates the OG-FSH group; -□- indicates the NG-FSH group.

In addition, recovery of the estrous cycle after transplantation on days 5, 8, 11, 14, and 17 was determined, and the results suggested that the number of mice with regular 4-day estrous cycles in the OG-FSH group was remarkably higher than the number of mice in the NG-FSH group (Figure 9).

## 4. Discussion

There is little damage to the primordial follicle during ovarian vitrification, but the damage from the primary to the mature follicles is caused by granulosa cell apoptosis [33]. In addition, most ovarian follicles, especially most primordial follicles, die as a result of ischemia/reperfusion injury in the early stages of transplantation [12]. Therefore, antiapoptosis in ovarian vitrification and improving the blood supply in early ovarian transplantation are two main issues for successful birth of offspring after ovarian vitrification. Indeed, the existence of apoptosis in ovarian cells during vitrification was further supported by our results from the detection of the apoptotic marker active caspase-3 and TUNEL analysis. Furthermore, our results suggested that administration with 0.3 IU/mL FSH significantly decreased the apoptosis of granulosa cells, and FSH might play a key role in preventing apoptosis during ovarian vitrification. Indeed, FSH suppressed apoptosis of ovarian granulosa cells [34] and promoted the follicle maturation [35]. Several studies have suggested that FSH is the earliest discovered hormone to stimulate the growth of the ovarian follicle, although complete knowledge of the milieu for follicular growth* in vivo* is still lacking. However, there is evidence that adequate levels of specific hormones are essential for the growth of healthy follicles* in vitro* [36]. The pituitary gonadotropin, FSH, may promote, if not be essential for, follicular growth in culture systems [37]. Moreover, FSH administration not only maintained the morphological integrity of caprine preantral follicles but also stimulated the activation of primordial follicles and the growth of activated follicles in culture [38]. Indeed, additional FSH treatment may stimulate excess primordial follicle development, thus accelerating follicular pool consumption, and administration of FSH after ovary transplantation might increase follicular pool consumption [39]; this may be attributed to the concentration of FSH; indeed, treatment with 25 IU FSH twice daily within 1 week following transplantation partly prevents primordial follicle loss in fresh and frozen-thawed tissues [40]; therefore, appropriate concentration of FSH plays an important role in ovarian vitrification.

Our results suggested that ovarian tissues treated with 0.3 IU/mL FSH during ovarian cryopreservation by vitrification remarkably protected the ovarian integrity and decreased the apoptotic rate. Consistent with our results, Friedman's results also suggested that FSH plays an important role in ovarian vitrified cryopreservation [41].

To explore the mechanism of FSH during ovarian cryopreservation by vitrification, the expression of Cx43 and Cx37 was evaluated, and these results suggested that the mRNA and protein levels of Cx43 and Cx37 in the OG-FSH group were significantly higher than those in the other groups. Moreover, the expression levels of Cx43 and Cx37 were upregulated by FSH during ovarian cryopreservation by vitrification. Indeed, Cx43 and Cx37 play important roles in the development and growth of follicles; and Cx43 and Cx37 were regulated by FSH [20, 42]. Thus, Cx43 and Cx37 represent downstream molecular targets of FSH during ovarian cryopreservation by vitrification, and FSH protects the intact ovary via the Cx43 and Cx37 signaling pathway. Indeed, previous studies have suggested that the expression of Cx43 and Cx37 in vitrified/warmed ovaries was lower than that in normal ovaries [42]. Furthermore, the signal transduction by connexin between granulosa cells and the oocytes was disturbed during vitrified cryopreservation [43].

Most ovarian follicles die from ischemia/reperfusion injury in the early stages of transplantation [12]; thus, neoangiogenesis and blood supply reconstruction were indispensable and increased around the transplanted ovary within 48 h [13, 14] to protect ovarian follicle survival. Therefore, the expression of VEGF and VEGFR2 was confirmed, and results suggested that the expression of VEGF and VEGFR2 in the OG-FSH group was significantly higher than that in the other groups. Furthermore, the expression of VEGF and VEGFR2 was regulated by FSH during ovarian cryopreservation by vitrification. Indeed, previous studies have suggested that VEGF and VEGFR2 were regulated by FSH [22, 44, 45], and upregulation of VEGF and VEGFR2 was beneficial both for the formation of new blood vessels during ovary transplantation and for increasing the rate of ovary transplantation. In addition, VEGF suppressed the apoptosis of ovarian granulosa cells [46]; thus, upregulation of VEGF and VEGFR2 plays an important role in antiapoptosis of the ovarian granulosa cell and the maintenance of the primordial follicle pool [47]. Indeed, VEGF protects the granulosa cell from apoptosis during the freeze-thaw process [48, 49]. Accordingly, ovarian vascular perfusion was performed at 48 h after successful transplantation, and these results suggested that neoangiogenesis in the OG-FSH and CG groups was remarkably increased compared with the NG-FSH group; these data were further validated by immunohistochemical staining with the endothelial cell markers CD31 and CD34; indeed, the expression of CD31 and CD34 was used to analyze microvessel density [49]. Moreover, the increased blood supply protected the primordial follicle from apoptosis under conditions of ischemia and hypoxia during the early stage of transplantation [49–51] and preserved the ovarian reserve. Indeed, more primordial follicles died from hypoxia and delayed revascularization than from freeze-thaw injuries [52].

Furthermore, the related index was quantified and analyzed, and the number of pregnant mice and the pregnancy rate in the OG-FSH group were remarkably increased compared with the NG-FSH group. In addition, the average litter size and the average litter size interval in the OG-FSH group were significantly higher than those in the NG-FSH group. In addition, the recovery of the estrous cycle in the OG-FSH group was shorter than that in the NG-FSH group. Thus, FSH remarkably increased the rate of ovarian transplantation, and VEGF and VEGFR2 might be downstream molecular signals of FSH during ovarian vitrified cryopreservation.

In conclusion, FSH administration during ovarian cryopreservation by vitrification maintained ovarian survival, specifically by maintaining ovarian integrity, decreasing ovarian apoptosis, increasing the blood supply of the transplanted ovary, and increasing the transplanted rate via upregulation of the expression of Cx43, Cx37, and VEGF/VEGFR2.

## Supplementary Material

Response: Checked and revised.
Supplementary 1: The preparation of medium including of basic medium, cultural solution, vitrified solution and warming solution.
Supplementary 2: The process of ovarian transplantation
a: The ovaries were heterotopically transplanted by the kidney capsule.
b: Ovaries were orthotopically transplanted into ovarian capsule.
Supplementary 3: The Ovaries from C57BL/6J strain mice (black) were orthotopically transplanted into C57BL/6J-Tyrc-2JJ albino strain mice (white), and then were mated with proven males of the same strain to induce natural pregnancy and birth.

## Figures and Tables

**Table 1:** Primer sequences used for real-time quantitative PCR.

Target gene	GenBank accession number	Primer sequence	Product size (bp)	Annealing temperature (°C)
GAPDH	XM_001476707.3	F: 5′-GGCATTGTGGAAGGGCTC-3′ R: 5′-GACCTTGCCCACAGCCTT-3′	156	60

Cx43	NM_010288.3	F: 5′-CGTCCCACGGAGAAAACCAT-3′ R: 5′-CGGTGGTGGCGTGGTAAG-3′	151	60

Cx37	NM_008120.3	F: 5′-AGCGGTTGCGGCAGAAAG-3′ R: 5′-CCCACGAATCCGAAGACGAC-3′	137	60

VEGF	NM_001025257.3	F: 5′-GTAACGATGAAGCCCTGGAGT-3′ R: 5′-TGTTCTGTCTTTCTTTGGTCTGC-3′	152	60

**Table 2:** The count of follicles on 48 h after ovarian transplantation.

Group	The percentage of primordial follicle	The percentage of primary follicle	The percentage of normal follicle	The percentage of atretic follicle
CG	36.9 ± 6.4^*∗*^	13.4 ± 5.4^*∗*^	50.3 ± 6.3^*∗*^	49.7 ± 6.3^*∗*^
NG-FSH	25.7 ± 4.1	2.7 ± 1.3	28.9 ± 5.1	71.5 ± 5.8

OG-FSH	34.9 ± 8.5^*∗*^	8.6 ± 1.6^*∗*^	43.5 ± 7.7^*∗*^	56.5 ± 7.7^*∗*^

*∗* indicates statistically significant difference in the same column.

**Table 3:** The counts of pregnancy rate and litter sizes of ovarian transplanted mice (6 months).

Group	The number of ovarian transplanted mice	The number of pregnant mice	Pregnancy rate (%)	Average of litter sizes	The average litter size interval (days)
CG	10	5	50	5.81 ± 1.28^*∗*^	43.58 ± 8.48^*∗*^
NG-FSH	10	3	30	3.75 ± 0.71	53.4 ± 11.89

OG-FSH	10	4	40	5.27 ± 1.10^*∗*^	40.5 ± 7.27^*∗*^

*∗* indicates statistically significant difference in the same column.

**Table 4:** The counts of pregnancy rate and litter sizes of ovarian transplanted mice (12 months).

Group	The number of ovarian transplanted mice	The number of pregnant mice	Pregnancy rate (%)	Average of litter sizes	The average litter size interval (days)
CG	10	5	50	5.65 ± 1.23^*∗*^	47.25 ± 10.99^*∗*^
NG-FSH	10	3	30	3.89 ± 0.78	54.5 ± 10.99

OG-FSH	10	4	40	4.47 ± 1.62^*∗*^	42 ± 9.50^*∗*^

*∗* indicates statistically significant difference in the same column.

## References

[1] Maltaris T., Beckmann M. W., Dittrich R. (2009). Review. Fertility preservation for young female cancer patients. *In Vitro*.

[2] Pieters R. (2010). Acute lymphoblastic leukaemia in children and adolescents: chance of cure now higher than 80%. *Nederlands Tijdschrift voor Geneeskunde*.

[3] Tanzler E., Morris C. G., Kirwan J. M., Amdur R. J., Mendenhall W. M. (2011). Outcomes of WHO grade i meningiomas receiving definitive or postoperative radiotherapy. *International Journal of Radiation Oncology Biology Physics*.

[4] Fasano G., Moffa F., Dechène J., Englert Y., Demeestere I. (2011). Vitrification of in vitro matured oocytes collected from antral follicles at the time of ovarian tissue cryopreservation. *Reproductive Biology and Endocrinology*.

[5] Chuai Y., Xu X., Wang A. (2012). Preservation of fertility in females treated for cancer. *International Journal of Biological Sciences*.

[6] Amorim C. A., Dolmans M.-M., David A. (2012). Vitrification and xenografting of human ovarian tissue. *Fertility and Sterility*.

[7] Amorim C. A., Jacobs S., Devireddy R. V. (2013). Successful vitrification and autografting of baboon (*Papio anubis*) ovarian tissue. *Human Reproduction*.

[8] Sheikhi M., Hultenby K., Niklasson B., Lundqvist M., Hovatta O. (2011). Clinical grade vitrification of human ovarian tissue: an ultrastructural analysis of follicles and stroma in vitrified tissue. *Human Reproduction*.

[9] Fathi R., Valojerdi M. R., Salehnia M. (2013). Effects of different cryoprotectant combinations on primordial follicle survivability and apoptosis incidence after vitrification of whole rat ovary. *Cryo-Letters*.

[10] Mazoochi T., Salehnia M., Valojerdi M. R., Mowla S. J. (2008). Morphologic, ultrastructural, and biochemical identification of apoptosis in vitrified-warmed mouse ovarian tissue. *Fertility and Sterility*.

[11] Salehnia M., Sheikhi M., Pourbeiranvand S., Lundqvist M. (2012). Apoptosis of human ovarian tissue is not increased by either vitrification or rapid cooling. *Reproductive BioMedicine Online*.

[12] Mahmoodi M., Mehranjani M. S., Shariatzadeh S. M. A., Eimani H., Shahverdi A. (2014). Effects of erythropoietin on ischemia, follicular survival, and ovarian function in ovarian grafts. *Reproduction*.

[13] Tang F., Zhang C., Wang X.-J., Wu D.-D., Zhou Y. (2010). Expression and clinical significance of VEGF and apoptosis in frozen-thawed mouse ovaries after transplantation. *Zhongguo Ying Yong Sheng Li Xue Za Zhi*.

[14] Wu D., Lei Y., Tong Y., Tang F., Qian Y., Zhou Y. (2010). Angiogenesis of the frozen-thawed human fetal ovarian tissue at the early stage after xenotransplantation and the positive effect of Salviae miltiorrhizae. *Anatomical Record*.

[15] Baird D. (2006). Role of FSH and LH in follicle development. *Journal de Gynecologie, Obstetrique et Biologie de la Reproduction*.

[16] Wang C., Roy S. K. (2009). Expression of bone morphogenetic protein receptor (BMPR) during perinatal ovary development and primordial follicle formation in the hamster: possible regulation by FSH. *Endocrinology*.

[17] Zhang C., Guo L., Zhu B. (2013). Effects of 3, 5, 3′-triiodothyronine (T3) and follicle stimulating hormone on apoptosis and proliferation of rat ovarian granulosa cells. *The Chinese journal of physiology*.

[18] Thomas F. H., Vanderhyden B. C. (2006). Oocyte-granulosa cell interactions during mouse follicular development: regulation of kit ligand expression and its role in oocyte growth. *Reproductive Biology and Endocrinology*.

[19] Krysko D. V., Mussche S., Leybaert L., D'Herde K. (2004). Gap junctional communication and connexin43 expression in relation to apoptotic cell death and survival of granulosa cells. *Journal of Histochemistry and Cytochemistry*.

[20] Johnson M. L., Redmer D. A., Reynolds L. P., Bilski J. J., Grazul-Bilskal A. T. (2002). Gap junctional intercellular communication of bovine granulosa and thecal cells from antral follicles: effects of luteinizing hormone and follicle-stimulating hormone. *Endocrine*.

[21] Doyle L. K., Walker C. A., Donadeu F. X. (2010). VEGF modulates the effects of gonadotropins in granulosa cells. *Domestic Animal Endocrinology*.

[22] Kuo S.-W., Ke F.-C., Chang G.-D., Lee M.-T., Hwang J.-J. (2011). Potential role of follicle-stimulating hormone (FSH) and transforming growth factor (TGF*β*1) in the regulation of ovarian angiogenesis. *Journal of Cellular Physiology*.

[23] Shin S.-Y., Lee H.-J., Ko D.-S., Lee H.-C., Park W. I. (2005). The regulators of VEGF expression in mouse ovaries. *Yonsei Medical Journal*.

[24] Wang Y., Chang Q., Sun J. (2012). Effects of HMG on revascularization and follicular survival in heterotopic autotransplants of mouse ovarian tissue. *Reproductive BioMedicine Online*.

[25] Zarnani A.-H., Moazzeni S.-M., Shokri F., Salehnia M., Jeddi Tehrani M. (2006). Analysis of endometrial myeloid and lymphoid dendritic cells during mouse estrous cycle. *Journal of Reproductive Immunology*.

[26] Ni H., Yu X.-J., Liu H.-J. (2009). Progesterone regulation of glutathione S-transferase Mu2 expression in mouse uterine luminal epithelium during preimplantation period. *Fertility and Sterility*.

[27] Yang Y., Jin Y., Lin P. (2013). The expression and localization of LRF in the female reproductive tract of cycling mice throughout the estrous cycle. *Journal of Immunoassay and Immunochemistry*.

[28] Yang Y., Lin P., Chen F. (2013). Luman recruiting factor regulates endoplasmic reticulum stress in mouse ovarian granulosa cell apoptosis. *Theriogenology*.

[29] Yang Y., Jin Y., Martyn A. C. (2013). Expression pattern implicates a potential role for luman recruitment factor in the process of implantation in uteri and development of preimplantation embryos in mice. *Journal of Reproduction and Development*.

[30] Marano J. E., Sun D., Zama A. M., Young W., Uzumcu M. (2008). Orthotopic transplantation of neonatal GFP rat ovary as experimental model to study ovarian development and toxicology. *Reproductive Toxicology*.

[31] Deng X., Zheng H., Yu X., et al (2009). Cryopreserved ovarian tissues can maintain a long-term function after heterotopic autotransplantation in rat. *Reproduction*.

[32] Tilly J. L. (2003). Ovarian follicle counts—not as simple as 1, 2, 3. *Reproductive Biology and Endocrinology*.

[33] Depalo R., Loverro G., Selvaggi L. (2002). In vitro maturation of primordial follicles after cryopreservation of human ovarian tissue: problems remain. *Medical and Pediatric Oncology*.

[34] Lin P., Rui R. (2010). Effects of follicular size and FSH on granulosa cell apoptosis and atresia in porcine antral follicles. *Molecular Reproduction and Development*.

[35] Han H., Silverman J. F., Santucci T. S. (2001). Vascular endothelial growth factor expression in stage I non-small cell lung cancer correlates with neoangiogenesis and a poor prognosis. *Annals of Surgical Oncology*.

[36] Picton H. M., Harris S. E., Muruvi W., Chambers E. L. (2008). The in vitro growth and maturation of follicles. *Reproduction*.

[37] Xu J., Bernuci M. P., Lawson M. S. (2010). Survival, growth, and maturation of secondary follicles from prepubertal, young, and older adult rhesus monkeys during encapsulated three-dimensional culture: effects of gonadotropins and insulin. *Reproduction*.

[38] Matos M. H. T., Lima-Verde I. B., Luque M. C. A. (2007). Essential role of follicle stimulating hormone in the maintenance of caprine preantral follicle viability in vitro. *Zygote*.

[39] Maltaris T., Beckmann M. W., Mueller A., Hoffmann I., Kohl J., Dittrich R. (2007). Significant loss of primordial follicles after prolonged gonadotropin stimulation in xenografts of cryopreserved human ovarian tissue in severe combined immunodeficient mice. *Fertility and Sterility*.

[40] von Schönfeldt V., Chandolia R., Ochsenkühn R., Nieschlag E., Kiesel L., Sonntag B. (2012). FSH prevents depletion of the resting follicle pool by promoting follicular number and morphology in fresh and cryopreserved primate ovarian tissues following xenografting. *Reproductive Biology and Endocrinology*.

[41] Friedman O., Orvieto R., Fisch B. (2012). Possible improvements in human ovarian grafting by various host and graft treatments. *Human Reproduction*.

[42] Sommersberg B., Bulling A., Salzer U. (2000). Gap junction communication and connexin 43 gene expression in a rat granulosa cell line: regulation by follicle-stimulating hormone. *Biology of Reproduction*.

[43] Navarro-Costa P., Correia S. C., Gouveia-Oliveira A. (2005). Effects of mouse ovarian tissue cryopreservation on granulosa cell-oocyte interaction. *Human Reproduction*.

[44] Alam H., Week J., Maizels E. (2009). Role of the phosphatidylinositol-3-kinase and extracellular regulated kinase pathways in the induction of hypoxia-inducible factor (HIF)-1 activity and the HIF-1 target vascular endothelial growth factor in ovarian granulosa cells in response to follicle-stimulating hormone. *Endocrinology*.

[45] Huang Y., Hua K., Zhou X. (2008). Activation of the PI3K/AKT pathway mediates FSH-stimulated VEGF expression in ovarian serous cystadenocarcinoma. *Cell Research*.

[46] Kosaka N., Sudo N., Miyamoto A., Shimizu T. (2007). Vascular endothelial growth factor (VEGF) suppresses ovarian granulosa cell apoptosis in vitro. *Biochemical and Biophysical Research Communications*.

[47] Roberts A. E., Arbogast L. K., Friedman C. I., Cohn D. E., Kaumaya P. T., Danforth D. R. (2007). Neutralization of endogenous vascular endothelial growth factor depletes primordial follicles in the mouse ovary. *Biology of Reproduction*.

[48] Shin S.-Y., Lee J.-Y., Lee E. (2006). Protective effect of vascular endothelial growth factor (VEGF) in frozen-thawed granulosa cells is mediated by inhibition of apoptosis. *European Journal of Obstetrics Gynecology and Reproductive Biology*.

[49] Irusta G., Abramovich D., Parborell F., Tesone M. (2010). Direct survival role of vascular endothelial growth factor (VEGF) on rat ovarian follicular cells. *Molecular and Cellular Endocrinology*.

[50] Lee R. K.-K., Ho H.-Y., Yu S.-L., Lu C.-H. (2005). Blastocyst development after cryopreservation and subcutaneous transplantation of mouse ovarian tissue. *Journal of Assisted Reproduction and Genetics*.

[51] Israely T., Nevo N., Harmelin A., Neeman M., Tsafriri A. (2006). Reducing ischaemic damage in rodent ovarian xenografts transplanted into granulation tissue. *Human Reproduction*.

[52] Lee R. K.-K., Li S.-H., Lu C.-H., Ho H.-Y., Chen Y.-J., Yeh H.-I. (2008). Abnormally low expression of connexin 37 and connexin 43 in subcutaneously transplanted cryopreserved mouse ovarian tissue. *Journal of Assisted Reproduction and Genetics*.

