# Supplementary material for: The Increased Expression of Connexin and VEGF in Mouse Ovarian Tissue Vitrification by Follicle Stimulating Hormone
Source: Biomed Res Int. 2015 Oct 11;2015:397264. doi: 10.1155/2015/397264 (PMC4620037; doi:10.1155/2015/397264)

Supplemental. 1

1. The preparation of basic medium (1000 ml)

Ingredients	Quantity
DMEM	5 g
F12	5.3 g
Streptomycin sulfate	0.1 g
Hepes	2.38 g
$NaHCO_3$	2.388 g
DMSO(dimethyl sulfoxide)	10 ml
Penicillin	150 IU
5.6% $NaHCO_3$	Adjust pH to 7.1-7.4
Triple distilled water	Added into total volume with 1000 ml

2. The preparation of cultural solution (50 ml)

12% BSA (bovine serum albumin) 5ml + basic medium 45ml

3. The preparation of vitrified solution

(1) Pre-equilibration solution:1.5 mol/L (50 ml)

EG (ethylene glycol) 8.36 ml+12% BSA 10 ml + basic medium 31.64 ml

(2) vitrified medium: 5.5 mol/L EG+0.5 mol/L sucrose+30% saccharosan (50 ml)

EG 15.3 ml+ sucrose 8.56 g+ saccharosan 15 g+12% BSA 10 ml+ basic medium 24.7 ml

4. Warming solution

(1) 0.5mol/L Thawing solution (50ml): sucrose 8.56 g+12% BSA 10 ml+ basic medium 40 ml

(2) 0.25mol/L Thawing solution (50ml): 0.5mol/L Thawing solution 25ml + basic medium 25ml

(3) 0.125mol/L Thawing solution (50ml): 0.25mol/L Thawing solution 25ml + basic

medium 25ml

Supplemental. 2

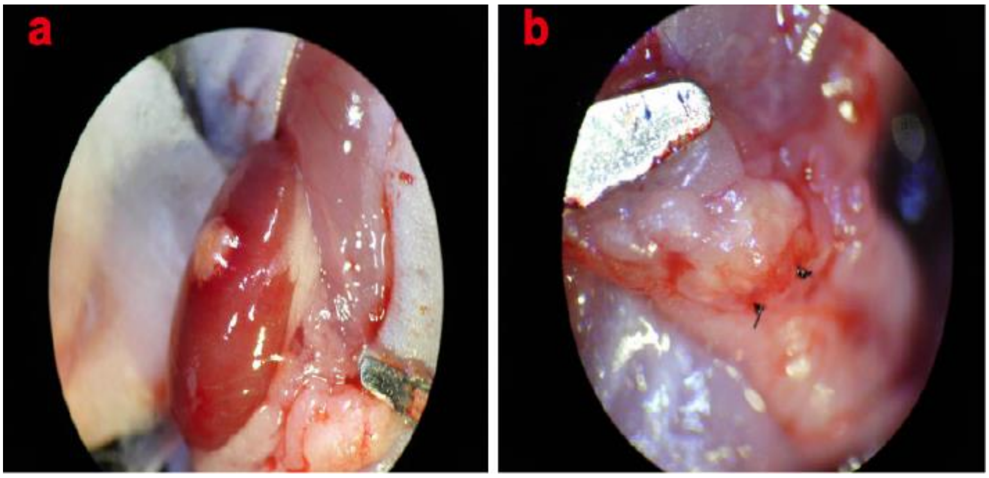

Supplemental. 3

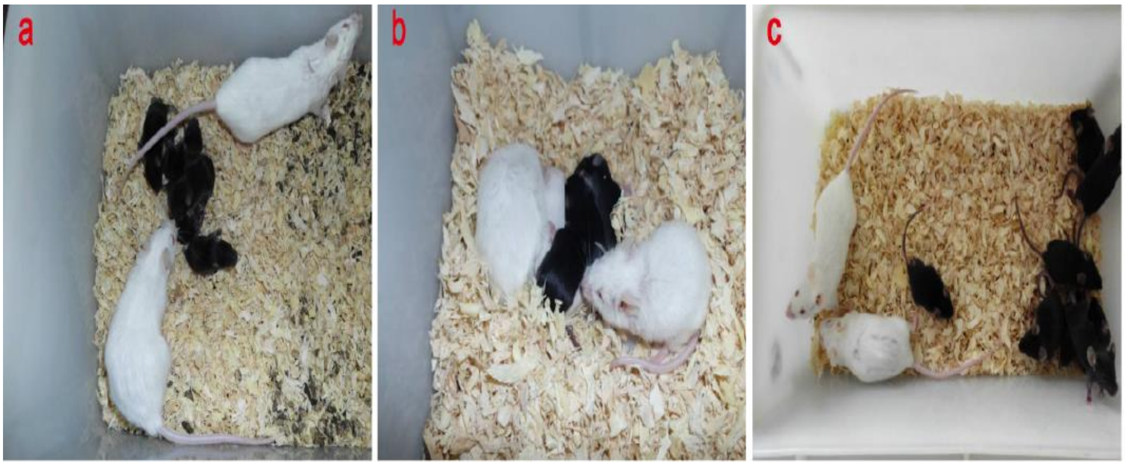

Supplement: Supplementary file 1 — Response: Checked and revised. Supplementary 1: The preparation of medium including of basic medium, cultural solution, vitrified solution and warming solution. Supplementary 2: The process of ovarian transplantation a: The ovaries were heterotopically transplanted by the kidney capsule. b: Ovaries were orthotopically transplanted into ovarian capsule. Supplementary 3: The Ovaries from C57BL/6J strain mice (black) were orthotopically transplanted into C57BL/6J-Tyrc-2JJ albino strain mice (white), and then were mated with proven males of the same strain to induce natural pregnancy and birth. [file 397264.f1.pdf]
